# The Effect of Pan-Retinal Photocoagulation on Central Macular Thickness in a Sample of Iraqi Patients With Proliferative Diabetic Retinopathy

**DOI:** 10.7759/cureus.67616

**Published:** 2024-08-23

**Authors:** Mohammad A Abbas, Ahmed S Al-Wassit, Mustafa Ismail

**Affiliations:** 1 Surgery, Ophthalmology Unit, Ghazi Al-Hariri for Surgical Specialties Hospital, Medical City Complex, Baghdad, IRQ; 2 Surgery, College of Medicine, University of Baghdad, Baghdad, IRQ; 3 Surgery, Baghdad Teaching Hospital, Baghdad, IRQ

**Keywords:** iraqi patients, central macular thickness, pan-retinal photocoagulation, optical coherence tomography, proliferative diabetic retinopathy

## Abstract

Objective: To evaluate the effect of pan-retinal photocoagulation (PRP) on central macular thickness (CMT) in a sample of Iraqi patients with proliferative diabetic retinopathy (PDR).

Methods: This prospective study was conducted at Ghazi Al-Hariri for Surgical Specialties Hospital, Baghdad, from March 2024 to May 2024. A total of 24 eyes from 18 treatment-naive PDR patients with no previous diabetic macular edema (DME) were enrolled. Each eye received PRP in two sessions, one week apart, using the Nidek GYC 500 laser system. CMT was measured at baseline and four weeks after the second PRP session using the Topcon DRI Triton Plus optical coherence tomography (OCT). Statistical analyses, including paired t-tests and Shapiro-Wilk tests for normality, were performed to evaluate changes in CMT.

Results: The mean CMT increased from 258.4 ± 30.7 microns at baseline to 269.9 ± 36.8 microns post PRP, with a mean increase of 11.5 ± 26.3 microns. This increase was statistically significant (p = 0.042). The Shapiro-Wilk test confirmed that the data were approximately normally distributed both before (W = 0.960, p = 0.445) and after (W = 0.931, p = 0.103) PRP treatment.

Conclusion: PRP significantly increases CMT in PDR patients, although no additional treatment for macular edema was necessary. These findings align with previous studies, suggesting that PRP-induced macular thickening is a common outcome. Further research is recommended to explore long-term effects and potential mitigation strategies.

## Introduction

Diabetic retinopathy (DR) is one of the leading causes of adult blindness between ages 25 and 74. The increasing global incidence of diabetes very much magnifies it [1]. When it becomes proliferative diabetic retinopathy (PDR), there is a high potential for extreme loss of vision. Pan-retinal photocoagulation (PRP), a treatment that now forms the mainstay in the management of PDR, is effective through decreasing the production of vascular endothelial growth factor (VEGF) by ablating ischemic regions of the retina and thereby preventing neovascularization.

Despite the effectiveness of PRP, it has been associated with complications such as visual field loss, retinal detachment, and macular edema. Macular edema, in particular, is a significant concern as it can lead to further vision impairment. Previous studies have reported varying effects of PRP on central macular thickness (CMT), with some indicating no significant changes and others showing increases in CMT [2-5]. 

Alternative treatments have developed to counter these pitfalls, and one of them is the introduction of intravitreal injections of anti-VEGF agents like bevacizumab and ranibizumab, which directly inhibit VEGF and seem to reduce macular edema with encouraging results in visual acuity improvement. Another option represented by corticosteroids, which reduce inflammation and, hence, vascular permeability, are delivered via intravitreal implants. However, these alternatives bear their problems as well, among which are intraocular pressure increase and cataract formation. The pathophysiology of DR is a complex interplay between metabolic and biochemical changes that are induced by hyperglycemia and cause damage to the retinal microvasculature. Chronic hyperglycemia results in an accumulation of advanced glycation end-products, oxidative stress, and inflammation, all of which contribute to breakdown of the blood-retinal barrier, thickening of the capillary basement membrane, and loss of pericytes. These changes ultimately lead to retinal ischemia and neovascularization, two hallmarks of PDR [2].

PRP occupies the central position in the management of PDR due to its effectiveness in reducing neovascularization. However, PRP has been reported to induce inflammatory responses that may increase CMT [2,3]. An anticipated rise in CMT following PRP will, therefore, have important implications for visual outcome as well as pose a risk of macular edema. Knowing if and to what degree this increase has occurred is important in interpreting the clinical significance of these measures and in guiding treatment decisions. This study aims at quantifying these changes and exploring their relevance with respect to existing treatment guidelines to provide data that may help inform strategies for the management of patients. We hypothesize that there will be an increase in CMT with PRP since it is itself an inciting incident of inflammation. It aims to quantify the change and its clinical significance as assessed in this study, in order to provide insights that could be used perhaps toward treatment guidelines to further underscore the importance of close follow-up when complications arise.

The burden of diabetes and its complications is increasing worldwide, including in Iraq. Hitherto, not enough research has been done emphasizing the Iraqi population on the outcome of PRP treatment in PDR. These data are essential, with this study reporting on CMT outcomes in a sample of Iraqi patients with PDR receiving PRP treatment; they significantly contribute to the existing literature, which is of great public health need in Iraq.

## Materials and methods

Study design

This study was done in the Ghazi Al-Hariri for Surgical Specialties Hospital, Medical City Complex, Baghdad, from March 2024 to May 2024. Written informed consent was collected from the participants before enrolling in the study.

Main Objective

It aimed to evaluate the effect of PRP on central CMT among PDR patients. Furthermore, The Institutional Review Board of the College of Medicine, University of Baghdad approved this research.

Study population

There were 18 cases in total: eight males and ten females, i.e., 24 eyes with PDR.

A thorough baseline examination was conducted for each participant, including visual acuity measurement using the Snellen chart, slit-lamp biomicroscopy to examine the anterior segment and vitreous, and a fundus examination using a +90D lens to assess the retina and optic nerve. Additionally, optical coherence tomography (OCT) imaging was performed to obtain detailed cross-sectional images of the macula. This comprehensive assessment ensured accurate diagnosis and confirmed the eligibility of participants for the study.

Inclusion Criteria

As part of the inclusion criteria, all of these eyes were treatment-naive, there was no previous history of DME or any other treatments for the retina in any of these eyes, there was a confirmed diagnosis of PDR by fundus examination, and a minimum duration of diabetes of 10 years. Exclusion criteria included previous ocular surgeries, co-existing retinal diseases like retinal vein occlusion and age-related macular degeneration, and systemic conditions that might affect the study endpoints, for example, uncontrolled hypertension and recent major surgery. Participants were recruited from the Ophthalmology Unit at Ghazi Al-Hariri for Surgical Specialties Hospital. This study included 18 participants: ten female and eight male, with their average age being 56.7 ± 8.3 years. The average known duration of diabetes was 12.5 ± 4.7 years, whereas all had PDR of severe grade according to the International Clinical Diabetic Retinopathy Disease Severity Scale.

The computerized randomization of follow-up measurement timings randomized patients into two groups. Participants and the OCT scanning technicians were not blinded for the treatment but were blinded for the adjustment of the camera settings; analysis of the data was performed by an independent biostatistician, blinded to treatment assignment.

Image acquisition protocol

Imaging was performed using the Topcon DRI Triton Plus OCT. Baseline OCT images were taken before the first session of treatment involving PRP, and follow-up images were taken four weeks after the administration of PRP. Pre- and post-treatment imaging involved scanning a 6x6 mm macular cube, centered at the fovea so as to ensure that the data collected is consistent. The OCT used in this experiment provides images with very high resolution, and its axial resolution is about 8 μm. All scans were performed by one experienced operator in the area to minimize variability of results.

CMT was measured using the Topcon DRI Triton Plus OCT system. Macular layer measurements through automated segmentation to promote consistency and accuracy were performed, with manual adjustments if necessary by the trained operator.

Treatment protocol

Each eye received PRP with a Nidek GYC 500 laser system (Nidek Co., Ltd., Gamagori, Japan) [6]. The Nidek GYC 500 laser system is a highly regarded laser photocoagulator frequently used in ophthalmology for retinal photocoagulation procedures. It features a green laser operating at a 532 nm wavelength, making it exceptionally effective for retinal treatments due to its optimal absorption by hemoglobin and melanin. This system provides high precision and control, which is crucial for the targeted treatment of retinal conditions such as DR, retinal vein occlusion, and retinal tears or detachments. Additionally, the Nidek GYC 500 is remarkably versatile, compatible with various delivery devices, including slit lamp adapters, endoprobes, and laser indirect ophthalmoscopes [6].

The PRP treatment was a minimum of 3,000 spots, and these were given in two sessions one week apart. To keep the parameters constant, the laser settings were standardized during all treatments. The power used was 200-400 milliwatts; the pulse duration was 100 milliseconds to reach moderate retinal burns. Each session targeted the peripheral retina to reduce ischemic territories and delay VEGF production. An eye doctor administered the treatments to standardize the treatment procedure.

Outcomes

CMT was evaluated using Topcon DRI Triton Plus OCT. Imaging with OCT was obtained at two different intervals, which included before the PRP therapy (baseline) and one month after the second PRP. The scanning protocol used for all participants was a 6x6 mm macular cube centered at the fovea to obtain detailed cross-sectional images of the macula. One experienced operator performed all OCT scans to avoid inter-operator variability. The primary outcome measure in this study was a change in CMT from baseline to four weeks post-PRP.

Data collection

**Figure 1:**
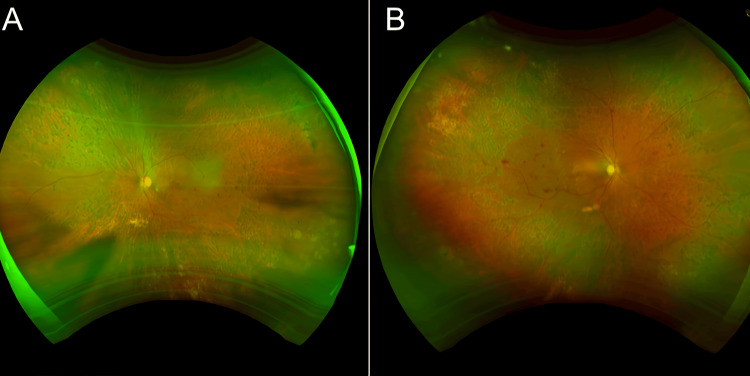
Fundus photographs of patients with PDR status post PRP one month prior (A): Fundus photograph of the left eye of a 70-year-old male with PDR, taken one month post-PRP. The laser treatment was done according to the DRS protocol, which is a standardized grid pattern of laser spots throughout the retina. Although empty spaces around the superior nasal arcade are visible, they were intentionally preserved to avoid any potential damage in sensitive areas such as optic nerve fibers. These spaces illustrate an aspect of treatment variation that can complicate, even if it is intended to be tailored to the specific retinal condition of the patient, the interpretation of the consistency of treatment outcomes. (B): Fundus photograph of the right eye of a 49-year-old male immediately post PRP for PDR. There are extensive laser photocoagulation marks, manifesting as multiple well-defined whitish lesions distributed over both the peripheral and central retina. The optic disc is within normal limits with clear margins. Relatively, the macular area is spared from significant hemorrhages or exudates PDR:  Proliferative diabetic retinopathy; PRP: Pan-retinal photocoagulation; DRS: Diabetic retinopathy study

**Figure 2:**
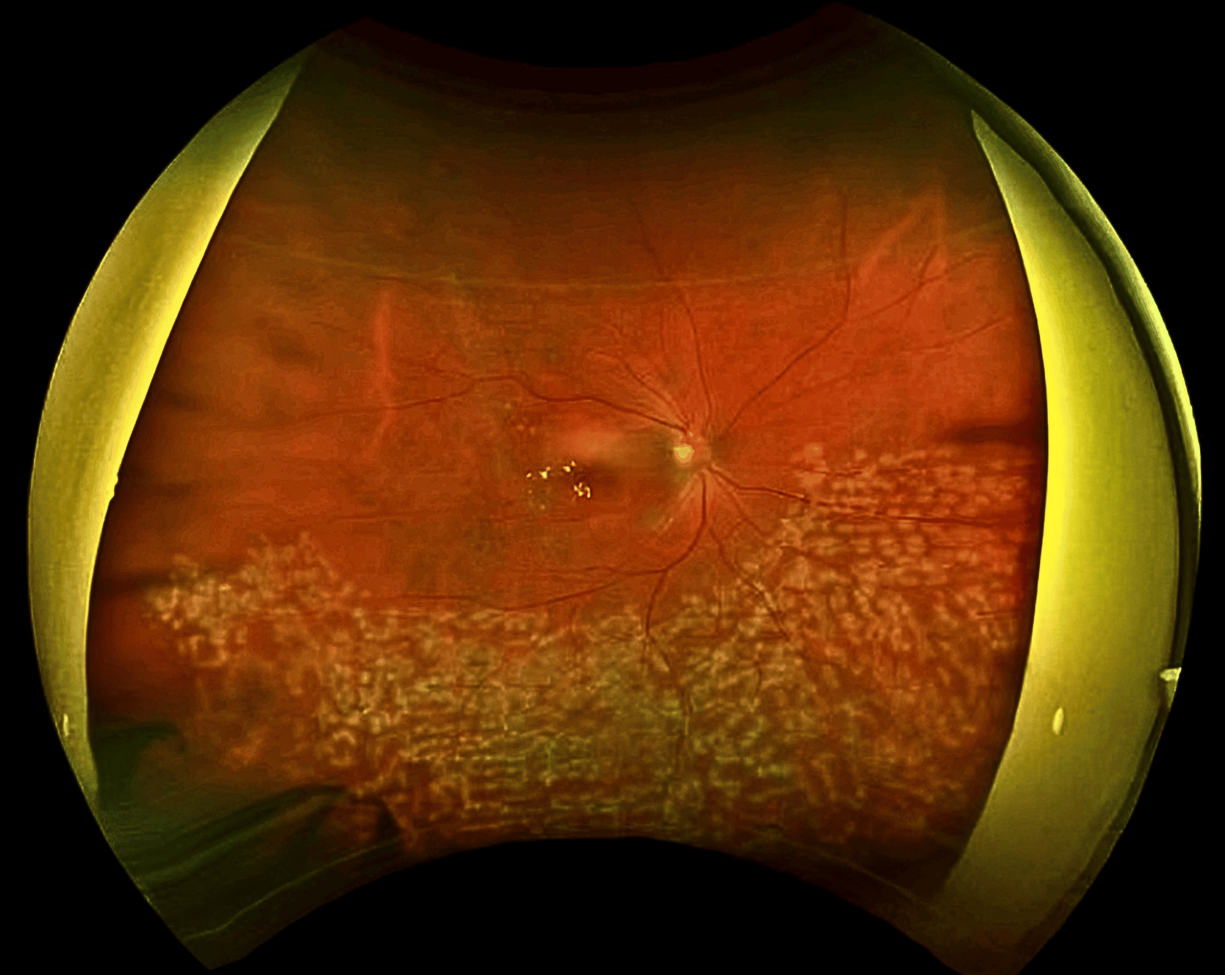
A wide-angle fundus photograph of a 49-year-old male immediately post PRP for PDR This is a wide-angle fundus photo of a 49-year-old male immediately post-first session of PRP for PDR. The image shows the multiple well-demarcated whitish spots all over large peripheral and posterior poles with extensive laser photocoagulation. The optic disc is well-perfused with clear margins, and the macula appears relatively spared from significant hemorrhages or exudates. PRP: Pan-retinal photocoagulation; PDR: Proliferative diabetic retinopathy

**Figure 3:**
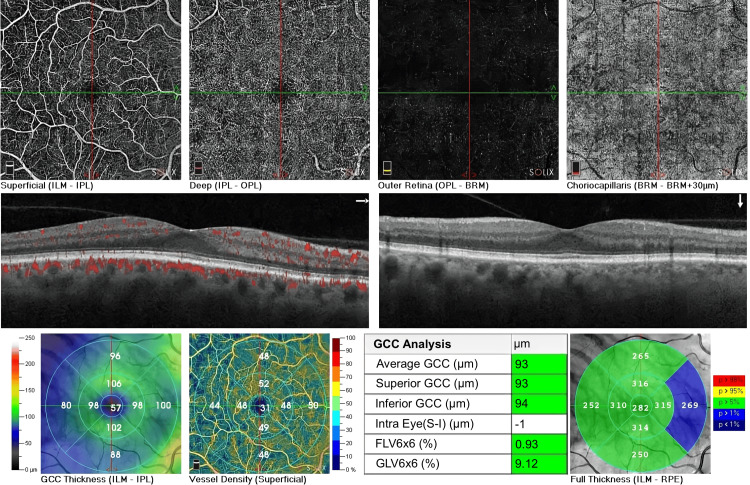
OCTA scans of the right eye (OD) of an adult, illustrating various retinal layers and measurements The OCTA scan, with a quality score of 8/10, illustrates the superficial (ILM-IPL), deep (IPL-OPL), outer retina (OPL-BRM), and choriocapillaris (BRM-BRM+30µm) layers. The superficial layer shows clear vasculature, the deeper layers are uniform, and the choriocapillaris remains intact with no signs of ischemia. The cross-sectional OCT B-scan with an angiography overlay displays well-organized retinal layers with areas of blood flow marked in red. The GCC thickness map reveals a central measurement of 57 µm, and the vessel density map shows a central density of 31%. GCC analysis indicates an average thickness of 93 µm, while the full retinal thickness map shows a central thickness of 282 µm. OCTA: Optical coherence tomography angiography; OCT: Optical coherence tomography; ILM-IPL: Inner limiting membrane to inner plexiform layer; IPL-OPL: Inner plexiform layer to outer plexiform layer; OPL-BRM: Outer plexiform layer to Bruch's membrane; BRM-BRM+30µm: Bruch's membrane to Bruch's membrane plus 30µm; GCC: Ganglion cell complex

Baseline data collected for all participants included age, gender, duration of diabetes, and baseline CMT. At four weeks after the second application of PRP, data follow-up was carried out to find out changes in CMT (Figures 1-3). Any complications and adverse events associated with the PRP treatment such as retinal detachment, vitreous hemorrhage, or high vision loss were noted. Data entry was done in a secure database in which two independent researchers cross-checked the recorded data for accuracy.

Statistical analysis

For statistical analysis, Jamovi software was used to compare baseline measurements with follow-up CMT. For each group, CMT mean values and standard deviations were calculated. The Shapiro-Wilk test was used to test the normality of the data distribution. For evaluating the significance of changes in CMT, paired t-tests were done; the value was taken as significant if less than 0.05. Descriptive statistics in frequencies, percentages, means, and standard deviations were used for the summary of the demographic and clinical characteristics of the study participants. The effect size of the CMT change was also computed to determine the magnitude of these differences.

Ethics statement

The present study aims to to evaluate the effect of PRP on CMT in a sample of Iraqi patients with PDR. The ethical considerations of this research are of paramount significance, and we are fully dedicated to performing this study in strict adherence to the highest ethical standards. The Institutional Review Board of College of Medicine, University of Baghdad has examined and approved this research on February 12, 2024 (IRB approval number is 36). All participants are required to provide informed consent. Participants receive detailed information regarding the study's objectives, methods, possible hazards, and their entitlements, including the right to resign at any point without facing any consequences. Each component of participant data has undergone anonymization. The researchers assert that they have no conflicting interests. The study is conducted without any external funding.

## Results

Participant demographics

A total of 18 patients (10 females and 8 males) participated in the study, contributing 24 eyes for analysis. The mean age of the participants was 56.7 ± 8.3 years, with a range from 45 to 70 years. The average duration of diabetes among the participants was 12.5 ± 4.7 years. All patients were treatment-naive for DR, with no previous history of DME or any other retinal treatments (Table 1).

**Table 1:** Comprehensive demographic information

City	Occupation	Gender	Mean Age ± SD	Min. Age	Max. Age	Count
Anbar	Free worker	Male	34.0 ± 0.0	34	34	1
Baghdad	Free worker	Male	34.0 ± 7.0	27	41	2
Baghdad	Housewife	Female	27.5 ± 2.5	25	30	2
Baghdad	Student	Female	18.4 ± 5.0	12	22	5
Baghdad	Student	Male	13.0 ± 3.0	10	16	2
Baghdad	Taxi driver	Male	38.0 ± 0.0	38	38	1
Karbala	Teacher	Male	33.0 ± 0.0	33	33	1
Mosul	Housewife	Female	28.0 ± 1.0	27	29	2
Najaf	Free worker	Male	36.0 ± 0.0	36	36	1
Najaf	Housewife	Female	29.0 ± 0.0	29	29	1

Baseline CMT

At baseline, the mean CMT was 258.4 ± 30.7 microns. The CMT measurements ranged from 204 to 314 microns. The most frequently observed CMT value (mode) was 229 microns, noted in 12.5% of the eyes. The baseline CMT distribution was confirmed to be approximately normally distributed (Shapiro-Wilk W = 0.960, p = 0.445).

Post-PRP CMT

Four weeks after the second PRP session, the mean CMT increased to 269.9 ± 36.8 microns, with a range from 218 to 335 microns. This increase in CMT from baseline was statistically significant, with a mean increase of 11.5 ± 26.3 microns (p = 0.042). The post-PRP CMT distribution was also approximately normally distributed (Shapiro-Wilk W = 0.931, p = 0.103).

Comparison of CMT before and after PRP

A paired t-test was conducted to compare the mean CMT before and after PRP treatment. The results indicated a statistically significant increase in CMT following PRP (p = 0.042). The effect size of the CMT change was computed to be 0.44, indicating a moderate effect of PRP on CMT (Table 2).

**Table 2:** CMT before and after PRP CMT: Central macular thickness; PRP: Pan-retinal photocoagulation

Measurement	N	Mean ± SD (Range)	Mode (µm)	Shapiro-Wilk W	Shapiro-Wilk p	Paired t-test p
Before PRP	24	258.4 ± 30.7 (204-314)	229.0	0.960	0.445	0.042
After PRP	24	269.9 ± 36.8 (218-335)	218.0	0.931	0.103	
Change (µm)	24	11.5 ± 26.3 (-32-83)	14.0	0.953	0.314	-

Complications and adverse events

No major complications such as retinal detachment or significant vision loss were observed during the study period. Minor complications included mild vitreous hemorrhage in two eyes (8.3%) and transient ocular discomfort in three eyes (12.5%). These complications resolved without additional intervention. No additional treatment for macular edema was required for any of the participants during the study period.

Additional observation

The study observed that the increase in CMT was not uniformly distributed among all participants. Some patients exhibited a more pronounced increase in CMT, while others showed minimal changes. The inflammatory response to laser treatment was hypothesized to be the primary cause of the observed increase in CMT. The laser-induced retinal burns may have triggered an inflammatory cascade, leading to increased vascular permeability and subsequent macular thickening. The absence of clinically significant macular edema (CSME) in the study participants suggests that the observed increase in CMT was subclinical and did not necessitate further therapeutic intervention.

The distribution of CMT measurements before PRP showed that the most common value (mode) was 229 microns, observed in 12.5% of the eyes. The range of CMT before PRP was from 204 to 314 microns, while after PRP, it ranged from 218 to 335 microns.

## Discussion

Our study aimed to evaluate the effect of PRP on CMT in a sample of Iraqi patients with PDR. The findings revealed a statistically significant increase in CMT four weeks after PRP treatment, with a mean increase of 11.5 microns. This is similar to other studies that reported an increase in CMT following PRP, indicating that post-treatment macular thickening is a common post-treatment complication.

Several studies have reported documented alterations in macular thickness following PRP treatment. Shahid et al. observed similar findings of an increase in CMT, as our findings suggest that the inflammatory response caused by laser therapy is accountable for this thickening [2]. Mukhtar et al., on the other hand, noted a significant increase in CMT post-PRP, referring to the pro-inflammatory cytokines and increased vascular permeability due to the laser procedure [3].

Additionally, our results support the conclusions of Soman et al., who demonstrated that PRP leads to macular thickening even in the absence of CSME at baseline [4]. This implies that the mechanical and thermal impacts of the laser can induce structural changes in the macula despite pre-existing edema.

One of the important considerations in interpreting our results is intra-treatment variability. Even within a standardized PRP procedure, some variability in terms of laser spot placement and intensity may occur, along with variability in patient response that could give way to different CMT outcomes. These may collectively explain the range of CMT increases seen in our study and provide impetus for the pursuit of more individualized treatment protocols that are better tailored to the anatomy of each retina and its pathologic unique condition. The accuracy of CMT measurements is crucial for assessing the effects of PRP. While we employed automated segmentation for OCT imaging, potential measurement errors or inconsistencies, such as variations in scan quality or operator adjustments, could impact the reliability of the results. We attempted to minimize these errors by using standardized imaging protocols and having an experienced operator conduct all scans.

Though the mean increase of CMT of 11.5 microns was found to be statistically significant, this should be viewed with respect to the clinical significance of an increase of this order. In relation to DR, there have been some studies showing that increases in CMT of around 10 microns or more actually tend to reduce visual acuity if the central macula is involved. This increase in our study was modest and did not meet or exceed the thresholds usually associated with CSME. As such, this observed CMT increase, although indicative of a post-treatment response, may be considered subclinical in nature and aligns with our findings that no participants required additional treatment for macular edema during the study period.

Preti et al. investigated the combined effect of PRP and intravitreal bevacizumab injections, having noted that while bevacizumab can take some of the macular thickening away, CMT still significantly increased with PRP alone [5]. Our study, focusing solely on PRP, provides additional evidence of the treatment's impact on macular thickness.

The study by Lee et al. reported a similar increase in macular thickness after PRP, which further added to the validation of our result and indicated the consistency of the effects of PRP on changes in different populations and settings [7]. Furthermore, Shimura et al. quantified these alterations and drew the point that there is a direct correlation between the area treated by PRP and the degree of macular thickening [8].

This increase in CMT brings multiple important clinical considerations to the table. Indeed, central-involving macular edema, evidenced by a significant increase in CMT, can compromise visual acuity and lead to problems with advanced daily living activities, such as reading, driving, and recognizing faces. These impairments to functioning underline the careful management required by patients receiving PRP, since an untreated or poorly managed macular edema would be expected to greatly reduce the quality of life for patients suffering from PDR. Keeping these findings in mind, there comes the role of anti-VEGF treatment. Administering anti-VEGF agents before or after PRP may help respond and control CMT, preventing vision loss. Pre-treatment with anti-VEGF would diminish baseline edema, and its post-PRP administration would control residual or exacerbated edema. Further studies should clarify the most appropriate timing and combination of PRP with anti-VEGF treatments for best outcomes in patients.

This may also include individualization of treatment entry protocols based on the response of the patient. Laser settings or the use of standard anti-VEGF in patients presenting at higher risk for macular edema can be beneficial. Regular follow-up is necessary for assessing changes in the CMT, and OCT imaging is used to monitor changes over time. It is a matter of keeping vigil on the additional interventions as necessary in preserving vision and patients' quality of life. Regular follow-up of PRP patients is very important, with emphasis on changes of CMT to detect any manifestations of worsening edema as early as possible. This should be followed by OCT imaging, and the treatment should be adjusted accordingly with further anti-VEGF injections or laser modifications in order to prevent loss of vision and preserve quality of life. From a clinical perspective, the importance of such an increase in CMT corresponds to a decreased visual outcome, a factor closely related to a patient's quality of life. In patients with PDR, visual difficulties are often already a problem, and further increases in CMT may increase that visual difficulty and perhaps lead to further problems with visual impairment and reduced independence. By implication, the results of the current study suggest that, when considering the best course of treatment, clinicians should focus not only on anatomical changes but also at the wider implications of these changes for patient well-being and quality of life.

Our study's findings are significant for the Iraqi population, because there is a shortage of information regarding PRP's effects in the Iraqi community. We provide valuable information about the local context by proving a statistically significant rise in CMT after PRP, showing the potential risk associated with this treatment. The knowledge of changes is essential for ophthalmologists of Iraq to know and manage effectively post-PRP changes [9-11].

This study has its limitations. The number of eyes in the study was relatively small at 24, with 18 patients; therefore, the findings might not be generalizable. In addition, four weeks of follow-up after PRP is a short duration to look for changes in CMT in the long run. This research design also lacked a comparison group, that is, untreated patients who could be used to lay the baseline for changes in macular thickness by treatment; this has made it difficult to attribute changes in macular thickness to the treatment per se. On the other hand, other influences, such as the variability of laser parameters and other inherent differences in individual patient responses to PRP, also would have been determining the results. Finally, this was a single-center study conducted in Iraq, and this could only generalize the findings to other populations [12-16].

In conclusion, our study adds to the increasing evidence that PRP treatment may result in increased CMT, and patients need a close observation [13,17-19]. Further research could be done on strategies to reduce this side effect, probably including adjuvant treatments such as anti-VEGFs to manage macular changes post PRP better.

## Conclusions

Our study has shown a significant increase in CMT following PRP in Iraqi patients with PDR. However, the increase was not clinically significant enough to mandate additional treatment of macular edema. The OCT findings in the study demonstrated central-involving macular edema, which, if it compromised vision, would usually need treatment. While our study was not designed to assess the change in CMT following PRP, the substantial increase in CMT witnessed in some participants suggests that close monitoring and possible intervention may be required in cases where vision is compromised. This supports similar previous work that deals with the common complication encountered in macular thickening post PRP. We highly recommend more studies on a larger scale with extended follow-ups to be conducted for better exposure regarding long-term effects on and exploring ways of mitigating this side effect.

## References

[1] Fathy C, Patel S, Sternberg P Jr, Kohanim S (2016). Disparities in adherence to screening guidelines for diabetic retinopathy in the United States: a comprehensive review and guide for future directions. Semin Ophthalmol.

[2] Shahid MH, Rashid F, Tauqeer S, Ali R, Farooq MT, Aleem N (2022). Change in central macular thickness on OCT after pan retinal photocoagulation. Pak J Med and Health Sci.

[3] Mukhtar A, Khan MS, Junejo M, Ishaq M, Akbar B (2016). Effect of pan retinal photocoagulation on central macular thickness and visual acuity in proliferative diabetic retinopathy. Pak J Med Sci.

[4] Soman M, Ganekal S, Nair U, Nair K (2012). Effect of panretinal photocoagulation on macular morphology and thickness in eyes with proliferative diabetic retinopathy without clinically significant macular edema. Clin Ophthalmol.

[5] Preti RC, Mutti A, Ferraz DA, Zacharias LC, Nakashima Y, Takahashi WY, Monteiro ML (2017). The effect of laser pan-retinal photocoagulation with or without intravitreal bevacizumab injections on the OCT-measured macular choroidal thickness of eyes with proliferative diabetic retinopathy. Clinics (Sao Paulo).

[6] (2024). GYC-500/500 Vixi Overview. https://usa.nidek.com/gyc-500/.

[7] Lee SB, Yun YJ, Kim SH, Kim JY (2010). Changes in macular thickness after panretinal photocoagulation in patients with severe diabetic retinopathy and no macular edema. Retina.

[8] Shimura M, Yasuda K, Nakazawa T, Kano T, Ohta S, Tamai M (2003). Quantifying alterations of macular thickness before and after panretinal photocoagulation in patients with severe diabetic retinopathy and good vision. Ophthalmology.

[9] Brown DM, Kaiser PK, Michels M (2006). Ranibizumab versus verteporfin for neovascular age-related macular degeneration. N Engl J Med.

[10] Elman MJ, Aiello LP, Beck RW (2010). Randomized trial evaluating ranibizumab plus prompt or deferred laser or triamcinolone plus prompt laser for diabetic macular edema. Ophthalmology.

[11] Gross JG, Glassman AR, Liu D (2018). Five-year outcomes of panretinal photocoagulation vs intravitreous ranibizumab for proliferative diabetic retinopathy: a randomized clinical trial. JAMA Ophthalmol.

[12] (1985). Photocoagulation for diabetic macular edema. Early treatment diabetic retinopathy study report number 1. Early treatment diabetic retinopathy Study research group. Arch Ophthalmol.

[13] Nguyen QD, Brown DM, Marcus DM (2012). Ranibizumab for diabetic macular edema: results from 2 phase III randomized trials: RISE and RIDE. Ophthalmology.

[14] Bressler SB, Qin H, Beck RW, Chalam KV, Kim JE, Melia M, Wells JA 3rd (2012). Factors associated with changes in visual acuity and central subfield thickness at 1 year after treatment for diabetic macular edema with ranibizumab. Arch Ophthalmol.

[15] Wells JA, Glassman AR, Ayala AR (2015). Aflibercept, bevacizumab, or ranibizumab for diabetic macular edema. N Engl J Med.

[16] Kern TS, Miller CM, Du Y (2007). Topical administration of nepafenac inhibits diabetes-induced retinal microvascular disease and underlying abnormalities of retinal metabolism and physiology. Diabetes.

[17] Brown DM, Nguyen QD, Marcus DM (2013). Long-term outcomes of ranibizumab therapy for diabetic macular edema: the 36-month results from two phase III trials: RISE and RIDE. Ophthalmology.

[18] Rechtman E, Danis RP, Pratt LM, Harris A (2004). Intravitreal triamcinolone with photodynamic therapy for subfoveal choroidal neovascularisation in age related macular degeneration. Br J Ophthalmol.

[19] Do DV, Schmidt-Erfurth U, Gonzalez VH (2011). The DA VINCI Study: phase 2 primary results of VEGF Trap-Eye in patients with diabetic macular edema. Ophthalmology.

